# Linker of nucleoskeleton and cytoskeleton complex proteins in cardiomyopathy

**DOI:** 10.1007/s12551-018-0431-6

**Published:** 2018-06-04

**Authors:** Matthew J. Stroud

**Affiliations:** British Heart Foundation Centre of Excellence, School of Cardiovascular Medicine and Sciences, King’s College London, London, UK

**Keywords:** LINC complex, Inner nuclear membrane, Outer nuclear membrane, Nuclear envelope, Nucleus, Cardiomyocyte, Cardiomyopathy, Laminopathy

## Abstract

The linker of nucleoskeleton and cytoskeleton (LINC) complex couples the nuclear lamina to the cytoskeleton. The LINC complex and its associated proteins play diverse roles in cells, ranging from genome organization, nuclear morphology, gene expression, to mechanical stability. The importance of a functional LINC complex is highlighted by the large number of mutations in genes encoding LINC complex proteins that lead to skeletal and cardiac myopathies. In this review, the structure, function, and interactions between components of the LINC complex will be described. Mutations that are known to cause cardiomyopathy in patients will be discussed alongside their respective mouse models. Furthermore, future challenges for the field and emerging technologies to investigate LINC complex function will be discussed.

## Introduction

**Fig. 1:**
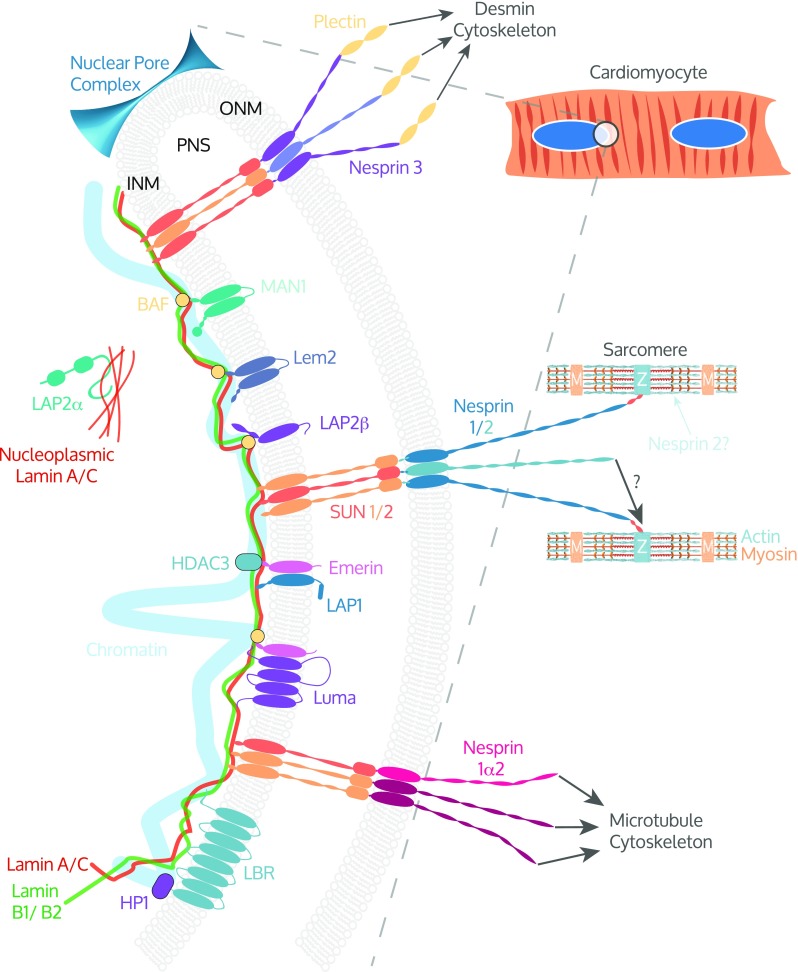
The cardiomyocyte LINC complex: Magnified view of cardiac myocyte nuclei (blue) in the circled region highlighting the nuclear envelope (NE) and linker between nucleoskeleton and cytoskeleton (LINC) complex. The LINC complex forms a continuous network of protein-protein interactions between the nuclear lamins; lamins A/C, B1, B2; and the various cytoskeletons. Inner nuclear membrane (INM) proteins SUN1 and SUN2 form heterotrimeric complexes that interact via their SUN domain with the KASH domain of the Nesprins that reside in the outer nuclear membrane (ONM). The giant isoforms of Nesprins 1 and 2 may directly link the NE to the sarcomere by interacting with the Z-disk (Z) or indirectly through intermediate binding partners(s). Nesprin 2 is reported at A/I junctions in the sarcomere. Nesprin 1α2 interacts with kinesin 1, thereby linking the NE to the microtubule cytoskeleton and Nesprin 3 indirectly links to the desmin intermediate filaments through plectin. Many other proteins are associated with the LINC complex that interact with lamins or SUN proteins including LAP2α, Emerin, MAN1, LEM2, Luma, and LAP1 and have been shown to play important roles in cardiomyocytes. Heterochromatin directly interacts with lamin A/C and indirectly with LEM domain proteins via barrier to autointegration factor (BAF). Emerin binds the histone modification enzyme histone deacetylase 3 (HDAC3). Lamin B receptor (LBR) interacts with lamin B and heterochromatin via heterochromatin protein 1 (HP1). LAP2α interacts with nucleoplasmic lamin A/C. PNS indicates perinuclear space and M, M-band

The nuclear envelope (NE) plays a critical role in dividing the cytoplasm from the nucleus. The NE is comprised of the inner nuclear membrane (INM) and the outer nuclear membrane (ONM), which is contiguous with the endoplasmic reticulum (Fig. 1). These membranes are separated by the perinuclear space (PNS) and are periodically joined by nuclear pore complexes (NPC) that allow bidirectional transport of macromolecules across the NE (Brohawn et al. 2009; Gorlich and Kutay 1999; Grossman et al. 2012). Underlying the INM is an interconnected meshwork of intermediate filaments collectively known as the nuclear lamina, which is made up of A-type and B-type lamins. Lamins play essential roles in maintaining nuclear structure, gene expression, and chromatin organization (Burke and Stewart 2013; Gerace and Tapia 2018). Furthermore, the nuclear lamina is functionally coupled to the cytoskeleton via connections mediated by INM and ONM proteins, termed the linker of nucleoskeleton and cytoskeleton (LINC) complex (Crisp et al. 2006; Sosa et al. 2013; Stroud et al. 2014) (Fig. 1). The LINC complex structurally supports the nucleus and plays a mechanosensory role to translate mechanical cues and alterations in the extracellular matrix into biochemical signals (Banerjee et al. 2014; Haque et al. 2006; Padmakumar et al. 2005; Sosa et al. 2012; Starr and Fridolfsson 2010; Starr and Han 2002; Swift et al. 2013), thereby allowing the cell to adapt to its surrounding environment by modulation of cytoskeleton organization, gene expression, nuclear organization, and structure (Jaalouk and Lammerding 2009; Lombardi and Lammerding 2011).

The importance of the LINC complex has been highlighted by the plethora of mutations in LINC complex-encoding genes that are associated with skeletal and cardiac myopathies, including dilated cardiomyopathy (DCM), arrhythmogenic cardiomyopathy (AC), and Emery-Dreifuss muscular dystrophy (EDMD) (Dellefave and McNally 2010; McNally and Mestroni 2017; Meinke and Schirmer 2016; Mejat and Misteli 2010; Mendez-Lopez and Worman 2012; Worman et al. 2010). Here, we will describe the roles of the LINC complex and its associated proteins that have been identified in the heart. The structural and functional interactions made between nuclear envelope spectrin repeat proteins (Nesprins), SUN proteins, Emerin, LAP1, LAP2, MAN1, LEM2, Luma, and nuclear lamins will be discussed, and mutations that lead to disease highlighted.

## Nuclear envelope spectrin repeat proteins

**Fig. 2:**
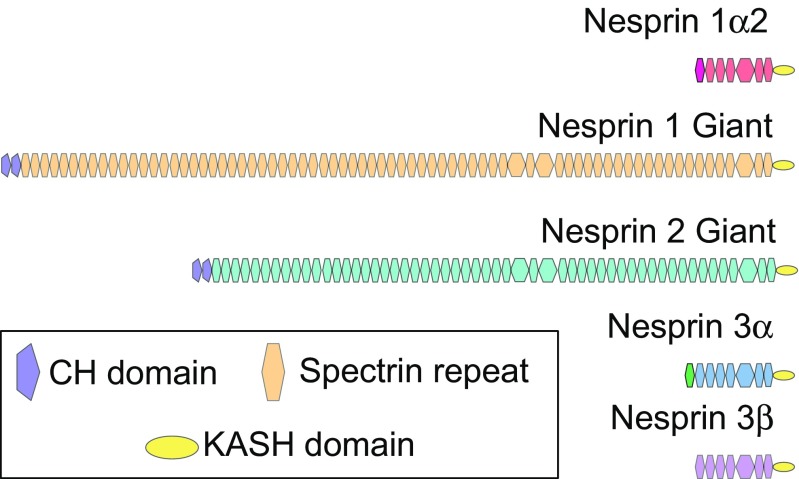
Nesprin family members and isoforms reported in cardiomyocytes. Nesprin 1α2 contains an isoform-specific spectrin repeat (pink) followed by 6 other spectrin repeats (red) and a KASH domain (yellow). Nesprin 1 giant contains N-terminal tandem calponin homology (CH) domains (purple) that bind actin, spectrin repeats (orange), and a C-terminal KASH domain. Nesprin 2 giant contains tandem CH domains, abutted to spectrin repeats (orange) and a KASH domain. Nesprin 3α is comprised of an isoform-specific spectrin repeat (green) that binds plectin followed by 7 spectrin repeats (blue) and a KASH domain. Nesprin 3β is organized similarly to Nesprin 3α, but the isoform-specific spectrin repeat found in 3α

Nesprins form the ONM component of the LINC complex that connects the nuclear lamina to the cytoskeleton (Apel et al. 2000; Crisp et al. 2006; Mislow et al. 2002b; Padmakumar et al. 2004; Shanahan et al. 1993; Zhang et al. 2001). The Nesprin family are comprised of four members (Nesprins 1–4) (Zhang et al. 2002), of which Nesprins 1, 2, and 3 have been detected in the heart (Banerjee et al. 2014; Ketema et al. 2013; Postel et al. 2011; Wilhelmsen et al. 2005) (Fig. 2).

## Nesprins 1 and 2

Nesprin 1 is alternatively named synaptic NE-1 (Syne-1) (Apel et al. 2000), Enaptin (Padmakumar et al. 2004), and myocyte NE protein-1 (Myne-1) (Mislow et al. 2002b) because of its simultaneously discovery by independent groups. Nesprin 2 is also known as Syne-2, or nucleus and actin connecting element (NUANCE) (Zhen et al. 2002). Many potential isoforms of Nesprins 1 and 2 exist, and they vary greatly in size due to alternative transcription initiation, termination, and RNA splicing of SYNE-1 and SYNE-2 genes, respectively (Rajgor et al. 2012). The largest, or giant (G), isoforms of Nesprins contain N-terminally paired calponin homology (CH) domains that bind actin, a spectrin repeat-containing rod domain, and a C-terminal Klarsicht, ANC-1, and Syne homology (KASH) domain that interacts with Sad1/UNC-84 (SUN) proteins (Padmakumar et al. 2004; Sosa et al. 2012) (Fig. 2). Other Nesprin isoforms exist that lack either the N-terminal CH domains, C-terminal KASH domain, or have a varying number of spectrin repeats (Warren et al. 2005).

Many antibodies have been raised against Nesprins 1 and 2 to different regions and domains (Potter et al. 2017; Randles et al. 2010; Razafsky and Hodzic 2015). Nesprin 1 epitopes have been reported at the NE and the Z-disc in both human and mouse skeletal and cardiac tissue (Apel et al. 2000; Holt et al. 2016; Mislow et al. 2002b; Nikolova-Krstevski et al. 2011; Zhang et al. 2002). Nesprin 2 epitopes have been reported at the NE, Z-disc, and A/I junction in human skeletal muscle, and at both NE and striations in mouse cardiac tissue (Banerjee et al. 2014; Zhang et al. 2005). However, it should be noted that the specificities of some of the antibodies described to date have not been thoroughly validated in suitable knockout animal models. Furthermore, the lack of isoform-specific sequences in the various Nesprin proteins make the generation of specific antibodies challenging.

In humans, several mutations in Nesprins 1 and 2 are associated with cardiomyopathy (Haskell et al. 2017; Puckelwartz et al. 2009, 2010; Zhang et al. 2007a; Zhou et al. 2017). However, the limited number of patients and small family pedigrees make it difficult to ascertain the critical Nesprin isoforms and whether the identified mutations in Nesprins 1 and 2 are the unequivocal cause of disease. Therefore, to understand the role of Nesprins 1 and 2 in the mammalian heart, several mouse models have been developed.

The initial Nesprin 1 mutant mouse models to study Nesprin 1 function either replaced the KASH domain (Nesprin 1^rKASH^) or deleted it (Nesprin 1^ΔKASH^) (Puckelwartz et al. 2009; Zhang et al. 2007b). Around half of Nesprin 1^rKASH^ mutant mice died perinatally due to respiratory failure and the surviving mice developed progressive muscle weakness and cardiomyopathy including cardiac conduction defects (Puckelwartz et al. 2009, 2010). The replacement of the KASH domain with 61 unrelated C-terminal amino acids prevents Nesprin 1 from binding SUN proteins, thereby disrupting the LINC complex. Notably, cardiomyocyte nuclei were elongated and heterochromatin levels were reduced in the Nesprin 1^rKASH^ mice. These results mirrored what had been shown previously in lamin A/C mutant cardiomyocytes and point to the importance of LINC complex integrity (Nikolova et al. 2004).

In contrast to Nesprin 1^rKASH^ mice, Nesprin 1^ΔKASH^ mutants were viable and able to breed as normal. However, mice in which Nesprin 2’s KASH domain was also deleted (Nesprin 1^ΔKASH^/Nesprin 2^ΔKASH^) died of respiratory failure 20 min after birth. The cardiac function of the Nesprin 1^ΔKASH^ mice was not reported, and it was unclear whether KASH domain-less Nesprin 1 isoforms were expressed in these mice that may have obscured the phenotype (Zhang et al. 2007b). Similarly to Nesprin 1^rKASH^, Nesprin 1^ΔKASH^ mice had altered nuclear positioning and shape in skeletal muscle, but these parameters were not reported in cardiomyocytes.

As noted above, Nesprin 1 isoforms lacking KASH domains exist; therefore, strategies to replace or delete the KASH domain may still be able to express these Nesprin 1 isoforms. To overcome this, Zhang et al. inserted LoxP sites either side of an exon in SYNE1 that is common to both KASH domain-containing and KASH domain-less isoforms to allow generation of Nesprin 1^−/−^ mice (Zhang et al. 2010). Akin to the Nesprin 1^rKASH^ model, Nesprin 1^−/−^ mice had reduced survival rates, exhibiting 60% perinatal lethality, growth restriction, and compromised exercise capacity. In agreement with the other studies of Nesprin 1 function, nuclear morphology and positioning in skeletal muscle were abnormal. However, no changes in cardiac contractile function were observed up to 12 months of age, suggesting that there may be some compensatory mechanism occurring in these mice.

Along these lines, Banerjee et al. reasoned that Nesprin 2 may be compensating for loss of Nesprin 1 in the heart and therefore took an approach to conditionally ablate Nesprin 1 expression in cardiomyocytes on a Nesprin 2-null background (hereafter called Nesprin 1/2 cKO) (Banerjee et al. 2014). Cardiac-specific deletion of Nesprin 1 was achieved by crossing Nkx2.5-Cre mice (McFadden et al. 2005) with the floxed Nesprin 1 mouse line used to generate Nesprin 1^−/−^ mice. Conditional ablation of Nesprin 1 or global deletion of Nesprin 2 alone did not alter cellular or cardiac function. Conversely, Nesprin 1/2 cKO mice developed cardiomyopathy at 10 weeks and displayed altered nuclear positioning, shape, and chromatin positioning. Furthermore, Nesprin 1/2 cKO mice had impaired gene expression changes in response to biomechanical load. These data suggest that Nesprins 1 and 2 play critical roles in normal cardiomyocyte function, thereby enabling the heart to adapt appropriately to mechanical demands. Failure to do so resulted in aberrant apoptosis, cardiac remodeling, fibrosis, and eventually heart failure, which are also observed in patients with mutations in Nesprins 1 and 2 (Haskell et al. 2017; Puckelwartz et al. 2010).

These studies highlight the power of using mouse models to understand mammalian cardiac function and the importance of a functional LINC complex. However, because the Nesprin 1 floxed allele (Zhang et al. 2010) would remove all Nesprin 1 isoforms, a key question that remained unanswered in the field was which of the Nesprin 1 isoforms was important for normal cardiac function?

Because Nesprin 1G and Nesprin 1α2 are the predominant isoforms of Nesprin 1 expressed in striated muscle (Duong et al. 2014; Padmakumar et al. 2004; Randles et al. 2010), we generated two novel mouse lines in which either the actin-binding domain of Nesprin 1G or the actin-binding domain of Nesprin 1α2 was ablated (Nesprin 1^ΔCH^ and Nesprin 1α2^−/−^, respectively) (Stroud et al. 2017). Surprisingly, Nesprin 1^ΔCH^ mice were born at Mendelian ratios, displayed no perinatal lethality nor loss in bodyweight compared to wildtype littermates. Furthermore, they lived normal lifespans, with no evidence of cardiac dysfunction up to 18 months of age. These data demonstrate that actin binding by Nesprin 1 is dispensable for viability and that the lack of actin binding in Nesprin 1G is not sufficient to explain the perinatal lethality observed in Nesprin 1^−/−^ mice. We therefore postulated that Nesprin 1α2 might be the critical isoform. Similarly to the Nesprin 1^−/−^ mice, Nesprin 1α2^−/−^ mice exhibited perinatal lethality and nuclear mispositioning in skeletal muscle fibers. Components of the LINC complex were largely unaffected, apart from SUN1, which was mislocalized from the NE and its levels slightly reduced, suggesting Nesprin 1α2 may preferentially bind to SUN1. Isolation of embryonic hearts from Nesprin 1α2^−/−^ at embryonic day 18.5 (E18.5) revealed normal heart weight/body weight ratios and histological analyses revealed no gross morphological defects. To understand whether Nesprin 1α2 was critical for normal heart development and function, cardiac-specific knockout mice were generated, but they exhibited normal heart function up to 14 months of age (unpublished data). These data are in agreement with previous findings that showed simultaneous deletion of Nesprins 1 and 2 is required to perturb cardiac function and suggest that Nesprin 2 expression in the heart may compensate for loss of Nesprin 1α2 (Banerjee et al. 2014).

It would be interesting to investigate whether Nesprin 1G and Nesprin 1α2 play similar or different roles in the heart by crossing the Nesprin 1^CH f/f^ and Nesprin 1α2^f/f^ with a cardiac-specific Cre on a Nesprin 2-null background. Alternatively, approaches to induce physiological or pathological cardiac hypertrophy in the Nesprin 1^ΔCH^ or Nesprin 1α2 cKO mice may be necessary to elicit a phenotype. Clearly, further work is needed to understand whether the cardiomyopathy-associated mutations in Nesprin 1 actually lead to cardiac dysfunction in mammalian hearts. With the advent of CRISPR/Cas9 technology to generate point mutations, knock-in mice that mimic human mutations will enable further exploration of Nesprins 1 and 2 function.

## Nesprin 3

In contrast to the many Nesprin 1 and 2 isoforms, Nesprin 3 exists as two isoforms; 3α (108 kDa) or 3β (99 kDa), with 3α the predominant isoform in the heart (Wilhelmsen et al. 2005) (Fig. 2). Both Nesprin 3 isoforms contain a C-terminal KASH domain that binds SUN 1/2 and localizes Nesprin 3 to the nuclear envelope in cardiac tissue (Ketema et al. 2013). The N-terminus of Nesprin 3α interacts with the ABD domain of plectin, which in turn interacts with intermediate filaments (Ketema et al. 2007).

In terms of Nesprin 3 function in vivo, there are currently no mutations described in Nesprin 3 associated with cardiomyopathy. Furthermore, ablation of Nesprin 3 expression in both zebrafish and mouse models resulted in no baseline phenotype (Ketema et al. 2013; Postel et al. 2011). It would be interesting to test whether Nesprin 3 KO mice have an abnormal cardiac response to stress because knockdown of Nesprin 3 in vitro leads to defects in cell migration and morphology (Khatau et al. 2012; Morgan et al. 2011; Petrie et al. 2014). However, these effects may be masked by the presence of Nesprins 1 and 2 and therefore may require ablation of all three Nesprin genes.

## SUN proteins

SUN proteins are named after their C-terminal SUN domain that was first identified in fission yeast protein Sad1 and *C. elegans* protein UNC-84 (Hagan and Yanagida 1995; Malone et al. 1999). In mammals, five SUN proteins have been identified (Starr and Fridolfsson 2010). SUN1 and SUN2 were cloned from human brain cDNA (Malone et al. 1999) and are ubiquitously expressed, whereas SUN3, SUN4, and SUN5 are tissue restricted in the testes (Frohnert et al. 2011; Gob et al. 2010; Xing et al. 2004). Importantly, SUN1 and SUN2 are expressed in the heart and skeletal muscle (Crisp et al. 2006; Puckelwartz et al. 2009; Zhang et al. 2010) and therefore will be discussed in more detail in this review. We refer readers elsewhere for a broader overview of SUN domain proteins in other cell types and tissues (Rothballer et al. 2013).

At the mRNA level, six different isoforms of SUN1 are potentially expressed in the heart (Gob et al. 2011; Nishioka et al. 2016). All of these isoforms are predicted to contain the SUN domain, stalk region, transmembrane (TM) domain, but vary in the length that the N-terminus protrudes into the nucleoplasm. There are potentially 6 and 12 isoforms of SUN2 that are expressed in mouse and human heart, respectively (www.ensembl.org). However, it remains to be elucidated whether the shorter SUN1 and SUN2 isoforms are translated into protein, and indeed whether they play different functional roles. Both SUN1 and SUN2 are type 2 membrane proteins and have similar domain architectures. The largest isoform of human SUN 1 is around 90 kDa and SUN2 is ~80 kDa. Overall, they are highly similar and share 64% homology (Haque et al. 2006). SUN1 and SUN2 comprise an N-terminal region that interacts with lamins inside the nucleus (Crisp et al. 2006; Haque et al. 2006; Nishioka et al. 2016; Sosa et al. 2013), followed by single TM domain that spans the INM. Adjacent to the TM domain is the ‘stalk’ region that is structurally made up of coiled-coil repeats necessary for trimerization (Hennen et al. 2018; Jahed et al. 2018; Sosa et al. 2012; Wang et al. 2012; Zhou et al. 2012). The most conserved region between SUN proteins is the C-terminal SUN domain that interacts with the KASH domain of Nesprins (Sosa et al. 2012; Wang et al. 2012; Zhou et al. 2012).

The integral nature of SUN proteins in the LINC complex makes it surprising that compared to other LINC components, relatively few mutations in SUN1 and SUN2 are associated with cardiomyopathy (Meinke et al. 2014). It appears that SUN proteins play a role as genetic modifiers because disease phenotypes are only observed in patients when mutations in SUN proteins are combined with other mutations in cardiomyopathy-associated genes. For example, the R453W mutation in LMNA normally results in a mild form of EDMD (Brown et al. 2001; Colomer et al. 2002; Fidzianska and Hausmanowa-Petrusewicz 2003; Raffaele Di Barletta et al. 2000; Voit et al. 1988; Vytopil et al. 2003); however, when combined with a W377C mutation in SUN1, it resulted in cardiac abnormalities followed by heart failure at age 34 (Meinke et al. 2014). Further evidence of their role as genetic modifiers comes from dystrophic (LMNA^−/−^) and progeric (LMNA^Δ9^) laminopathy mouse models, as well as from patient-derived fibroblasts. SUN1 was shown to be dramatically upregulated in these mouse models. Interestingly, ablation of SUN1 in both of these models extended lifespan and ameliorated the phenotypes observed (Chen et al. 2012). Specifically, cardiac function as assessed by ejection fraction was restored to near wildtype levels of ~ 70% in DKO mice compared to ~ 50% in LMNA^−/−^ mice. Furthermore, the noticeable increase of sarcoplasmic vacuoles and number of inflammatory cells in the LMNA^−/−^ mice were reduced in DKO mice. The underlying molecular mechanisms behind the pathogenesis are unknown, but the authors noted that the cytotoxicity may be due to the excessive accumulation of SUN1 in the Golgi apparatus.

In mice, global ablation of SUN1 expression resulted in sterility due to an essential role of SUN1 in gametogenesis (Ding et al. 2007); however, no changes in cardiac or skeletal muscle function were noted. Unlike SUN1 KO mice, SUN2 KO mice were normal presumably due to functional redundancy between SUN proteins (Lei et al. 2009). Conversely, global ablation of SUN1 and SUN2 resulted in perinatal lethality, likely because of respiratory defects. Interestingly, the perinatal lethality observed in these mice could be rescued by overexpressing SUN1 with a neural-specific promoter. Furthermore, it is intriguing that the DKO mice have a similar phenotype to the Nesprin 1α2 KO and Nesprin 1/2 DKO mice. Taken together, these data suggest that SUN1 may play unique, non-redundant roles in neurons and the reproductive organs. Whether SUN1 and SUN2 play essential, non-redundant roles in the heart remain unknown.

The generation of floxed alleles of these genes will enable targeted deletion of specific isoforms in specific tissues to further our understanding in cardiomyocytes. Furthermore, armed with the knowledge that SUN proteins likely play key roles as genetic modifiers, it would be interesting to sequence SUN1 and SUN2 genes in patients with cardiomyopathy in which other LINC complex mutations have been identified that may not fully explain the resulting phenotype. This may reveal clues as to why certain strong cardiomyopathy/EDMD phenotypes in the clinic are not well recapitulated in mouse models that are designed to mimic the exact mutation found in humans (Ozawa et al. 2006; Stroud et al. 2018).

## Emerin

Emerin is ubiquitously expressed in tissues and localizes to the NE in both cardiac and skeletal muscle (Manilal et al. 1996; Nagano et al. 1996; Stroud et al. 2018, 2017). Emerin is a type II membrane protein in which the nucleoplasmic N-terminus is adjacent to a TM domain that traverses the INM followed by a short luminal region that resides in the PNS (Manilal et al. 1996; Nagano et al. 1996). The interaction between Emerin and A-type lamins is thought to be important for its retention at the INM (Cartegni et al. 1997; Holaska et al. 2002; Manilal et al. 1996; Nagano et al. 1996). Other binding partners exist and include SUN1, SUN2, Nesprin 1α, HDAC3, and BAF, the latter two providing a link between Emerin and heterochromatin (Berk et al. 2014; Demmerle et al. 2012; Haque et al. 2010; Lee et al. 2001; Mislow et al. 2002a). For an extensive list of Emerin-binding partners, we refer the reader here (Berk et al. 2013).

At the cellular level, Emerin-null mouse embryonic fibroblasts (MEFs) have abnormal nuclear shape, altered NE plasticity, and impaired response to mechanical stimulation (Lammerding et al. 2005; Rowat et al. 2006). These data suggest that Emerin may regulate gene expression to enable the cell to adapt to mechanical load. In support of this, Emerin has been shown to indirectly regulate the localization of the mechanosignalling transcription factor megakaryoblastic leukaemia 1 (MKL1) by modulating actin dynamics (Ho et al. 2013). MKL1, also known as myocardin-related transcription factor a (MRTF-A), is a co-activator of serum response factor, which is a master regulator of cytoskeletal proteins such as vinculin and actin.

Mutations in Emerin lead to X-linked EDMD, cardiac conduction defects, DCM, and skeletal muscle defects (Bione et al. 1994, 1995; Nagano et al. 1996; Nigro et al. 1995; Vohanka et al. 2001; Yamada and Kobayashi 1996). For an extensive overview of disorders caused by Emerin mutations, we refer the reader to recent reviews (Astejada et al. 2007; Holaska 2008). The majority of these mutations are predicted to result in loss of Emerin expression (Manilal et al. 1996; Nagano et al. 1996). Therefore, Emerin KO mice were generated to study its function in the heart and skeletal muscle (Lammerding et al. 2005; Melcon et al. 2006; Ozawa et al. 2006; Stubenvoll et al. 2015). Surpisingly, Emerin-null mice have no overt skeletal myopathy and a mild atrioventricular conduction defect that is age-related (Melcon et al. 2006; Ozawa et al. 2006). Interestingly, a recent study revealed that Emerin KO mice have a mild, albeit significant reduction in ejection fraction that was observed using functional magnetic resonance imaging (MRI) at 12 weeks (Stubenvoll et al. 2015). Furthermore, Emerin KO mice challenged with transverse aortic constriction (TAC)-induced pressure overload showed a decline in ejection fraction and increase in end-systolic volume at 6 months after TAC compared to wildtype controls (Rockman et al. 1991; Stubenvoll et al. 2015). This study revealed that Emerin-null hearts contain an increased number of small cardiomyocytes that was likely due to aberrant β-catenin signaling during development, which resulted in an increase in cell proliferation at embryonic day 12.5 (E12.5). These data suggest that Emerin plays a role in the heart under baseline conditions and is necessary to cope with the consequences of pressure-induced cardiac overload.

Despite these conflicting findings about Emerin’s function in the heart, it is clear that Emerin-null mice do not develop a severe skeletal muscle myopathy as might be expected from patient data. To explain this difference, Shin et al. recently showed that the disparities observed between mouse and humans are likely due to the differential expression of Emerin between the two species. Specifically, Emerin levels in human skeletal muscle were found to be much higher than in the equivalent mouse tissue, suggesting that other protein(s) may be able to functionally compensate (Shin et al. 2013). Indeed, the authors found the expression levels of Emerin’s binding partner, lamina-associated polypeptide 1 (LAP1), were higher in mouse than in human. In line with LAP1 playing an important role in muscle, conditional ablation of LAP1 in mouse skeletal muscle (LAP1 scKO) led to muscular dystrophy resulting in premature death.

Furthermore, the authors wanted to understand whether there was a functional interaction between Emerin and LAP1, and therefore crossed LAP1 scKO with Emerin-null mice to generate LAP1 scKO/Emerin^−/y^ (herein named DKO). Strikingly, DKO mice had an exacerbated myopathy and their median survival was reduced to more than half compared to LAP1 scKO mice. These data suggest that Emerin and LAP1 both physically and functionally interact in skeletal muscle (Shin et al. 2013).

## Lamina-associated polypeptide 1

Lamina-associated polypeptide 1 (LAP1) consists of three isoforms (A, B, C) that are the result of alternative mRNA splicing of the TOR1AIP1 gene. Initially discovered in rat liver nuclear envelope extracts (Senior and Gerace 1988), they have recently been described in mouse cardiac and skeletal muscle (Shin et al. 2014, 2013). LAP1B and LAP1C are expressed in human hearts, with LAP1B thought to be the predominant isoform (Rebelo et al. 2015; Santos et al. 2014).

LAP1A, LAP1B, and LAP1C have predicted molecular weights of 75, 68, and 55 kDa, respectively (Senior and Gerace 1988). All LAP1 isoforms are type II membrane proteins. The N-terminus is located on the nucleoplasmic side of the INM and interacts with nuclear lamins and Emerin (Foisner and Gerace 1993; Shin et al. 2013). Emerin binding has been shown to be essential for normal skeletal muscle function (Shin et al. 2013). The C-terminus interacts with the Torsin AAA+ ATPase family members Torsin A, B, 2, and 3 (Goodchild and Dauer 2005; Jungwirth et al. 2010; Kim et al. 2010). Interestingly, global ablation of LAP1 was sufficient to exactly phenocopy both the nuclear blebbing and perinatal lethality observed in Torsin A KO mice, which suggests the interaction may have functional importance in neurons (Kim et al. 2010). Whether there is a similar functional interaction between the Torsin family member enriched in the heart, Torsin B, and LAP1 remains to be elucidated.

In humans, several mutations in the LAP1 gene are associated with cardiac defects (Dorboz et al. 2014; Kayman-Kurekci et al. 2014). For example, a nonsense frameshift mutation c.186delG in the TOR1AIP1 gene was identified in a three-generation family diagnosed with autosomal recessive limb-girdle muscular dystrophy with joint contractures (Kayman-Kurekci et al. 2014). Notably, one of the family members presented with reduced cardiac function as measured by echocardiography with an ejection fraction of 57% but had normal sinus rhythm as assessed by electrocardiogram analysis. Ultrastructural analysis of muscle biopsies revealed that the sarcomeres were intact, but the nuclei were fragmented and nuclear envelope disrupted. As expected, Western blot analysis of muscle tissue from the affected patient revealed the loss of the LAP1B band at 68 kDa, but noted the upregulation of a band at ~ 50 kDa. With the recent identification of LAP1C in humans (Santos et al. 2014), others have speculated that this ~ 50 kDa band might be LAP1C (Rebelo et al. 2015). Whether this band is indeed LAP1C remains to be determined. Nevertheless, it is apparently unable to completely rescue the phenotype caused by loss of LAP1B. It is also possible that the 50 kDa protein may partially compensate for the loss of LAP1B because deletion of all LAP1 isoforms in mice results in a much more severe form of skeletal myopathy with cardiac involvement (Shin et al. 2013). Further support for this hypothesis is from evidence of a missense mutation in LAP1 that changes glutamic acid 482 to alanine (E482A) and was identified in a patient with progressive dystonia, cerebellar atrophy, and DCM (Dorboz et al. 2014). Unlike the nonsense mutation c.186delG described above, the E482A mutation led to a more severe form of DCM that resulted in death at age 17. Western blot and immunofluorescence analysis of patient fibroblasts revealed a drastic reduction of all three LAP1 isoforms. Interestingly, the ultrastructure of the nuclear envelope in fibroblasts appeared normal. Whole exome sequencing was used to reveal this mutation, but it was unclear whether other mutations in genes that are strongly associated with DCM, such as LMNA (encoding lamin A/C), were investigated.

LAP1 function has also been investigated using mouse models (Kim et al. 2010; Shin et al. 2014, 2013). As mentioned above, deletion of all LAP1 isoforms in mouse striated muscle resulted in severe skeletal and cardiac myopathy (Shin et al. 2013). However, the specific role of LAP1 in the heart remained elusive. To investigate this, Shin et al. made use of their LAP1 floxed mice to generate a cardiomyocyte-specific knockout of LAP1 (herein referred to as LAP1 cKO) (Shin et al. 2014). LAP1 cKO mice were born at the expected Mendelian ratios and appeared overtly normal at 20 weeks; however, echocardiography revealed a significant reduction in cardiac function as measured by fractional shortening. Furthermore, levels of the fetal genes ANP and BNP as well as the pro-fibrotic markers collagen 1a1, collagen 1a2, and fibronectin 1 were all significantly upregulated in LAP1 cKO hearts. Mutant mouse models generated to study the function of LAP1’s interaction partners lamin A/C (LMNA^H222P/H222P^) and Emerin (EMD^−/−^) have previously shown activated extracellular signal-regulated kinase 1/2 (ERK1/2), c-Jun N-terminal kinase (JNK), and p38alpha branches of the mitogen-activated protein kinase (MAPK) signaling pathways (Muchir et al. 2007a, b). Therefore, the authors investigated whether these were affected in the LAP1 cKO mice. Interestingly, they found that ERK1/2 and JNK pathways were activated, but not p38alpha. The authors speculate that the extent of activation of these pathways may depend on the severity of the phenotype. For example, the LMNA^H222P/H222P^ mice (which develop significant DCM at 20 weeks) have all three pathways activated, whereas only ERK1/2 were activated in Emerin-null mice.

With regard to recent evidence from human data, suggesting that LAP1C might be upregulated and partially compensate for LAP1B loss, it would be interesting to generate LAP1 isoform-specific knockout mice. This would shed light on which LAP1 isoform(s) are critical for heart development and function.

## Lamina-associated polypeptide 2

Due to alternative splicing of the lamina-associated polypeptide 2 (LAP2) gene, there are six potential isoforms of LAP2. Five of which have been observed in both mouse and human hearts (LAP2α, LAP2β, LAP2γ, LAP2δ, LAP2ε) (Gotic et al. 2010; Harris et al. 1994; Taylor et al. 2005).

LAP2, also known as thymopoietin (TMPO), is one of the founding members of the LAP2, Emerin, and MAN1 (LEM) domain-containing family of proteins (Lin et al. 2000). All LAP2 isoforms contain an N-terminal LEM-like domain followed by the LEM domain that interacts with barrier to autointegration factor (BAF), which in turn binds to heterochromatin and double-stranded DNA (Montes de Oca et al. 2011). Whereas the N-termini of the LAP2 isoforms share high degrees of homology, their C-termini vary both in terms of length and the domains contained (Berger et al. 1996). Compared to the other isoforms of LAP2, LAP2α lacks a TM domain but contains an extended C-terminus that is important for its interaction with lamin A/C and mostly localizes to the nucleoplasm (Dechat et al. 1998, 2000). Conversely, LAP2β contains a TM domain and binds to B-type lamins at the nuclear envelope (Foisner and Gerace 1993; Furukawa et al. 1998). Presumably, the other TM domain-containing isoforms of LAP2 localize similarly to LAP2β.

LAP2α is the best characterized isoform of LAP2 and has been studied in both human and mouse hearts and will therefore be discussed in detail. In terms of LAP2α function, a missense mutation in LAP2α has been associated with a severe form of DCM that was diagnosed in two brothers at ages 33 and 22 (Taylor et al. 2005). The mutation identified changes arginine 690 to cysteine (R690C) in the C-terminus of LAP2α in a region that interacts with lamin A/C. Functional analysis of the R690C mutant in vitro revealed that the ability of LAP2α to interact with lamin A/C was reduced by around 50–75% compared to wildtype.

Since no patient biopsies were available, it was unclear whether LAP2α was expressed at normal levels in the myocardium. Therefore, to understand LAP2α’s function in vivo, loss of function mouse models were generated (Gotic et al. 2010; Naetar et al. 2008). Although the LAP2α^−/−^ mice were grossly indistinguishable from their littermates and lived normal lifespans, they had significantly reduced cardiac function as measured by fractional shortening at 10 weeks of age. LAP2α^−/−^ mice had normal heart weight:body weight ratios, but 18% of older mice developed extensive subendocardial fibrosis of the left ventricle. At the molecular level, master cardiac transcription factors MEF2c and GATA4 as well as their downstream targets were deregulated in LAP2α^−/−^ mice. LAP2α^−/−^ mice showed a blunted response to chronic infusion of isoproterenol to induce hypertrophy likely due to downregulation of the beta-adrenergic receptor β_2_AR. To further investigate the role of LAP2α in striated muscle cells (including cardiomyocytes), LAP2α floxed mice were crossed with mice expressing Cre recombinase under control of the muscle creatine kinase promoter. However, these mice did not develop systolic dysfunction, suggesting that LAP2α may play a more critical role during early stages of heart development. In support of this, LAP2α expression levels were higher during embryonic heart development (E12 and E14) and were downregulated at postnatal day 2 (Gotic et al. 2010).

Given the timing of LAP2α expression in embryonic cardiomyocytes, it would be interesting to ablate LAP2α expression using an earlier cardiomyocyte-specific Cre, such as XMLC2v that is expressed at E7.5 (Breckenridge et al. 2007). This will be important to understand whether the cardiac defects in the germline deleted LAP2α^−/−^ mice were cardiomyocyte-autonomous or due to loss of LAP2α expression in other cell types. In addition, the role of other LAP2 isoforms remains unexplored in terms of cardiac function.

## MAN1

Human MAN1, also known as LEM domain-containing protein 3 (Lemd3), is an 82.3 kDa INM protein that comprises an N-terminal LEM domain, two TM domains followed by MAN1-Src1-p C-terminal (MSC) domain and an RNA recognition motif (RRM) (Lin et al. 2000). MAN1 is ubiquitously expressed and has been found at the RNA level in human hearts (Lin et al. 2000). It has been shown to interact with lamin A/C, BAF, Smads 2 and 3, PPM1A (Smad2/3 phosphatase) and to regulate transforming growth factor β (TGF-β) and bone morphogenetic protein (BMP) signaling pathways through its interaction with Smads (Bourgeois et al. 2013; Cohen et al. 2007; Ishimura et al. 2006; Lin et al. 2005; Osada et al. 2003; Pan et al. 2005; Raju et al. 2003). Specifically, Smads 2 and 3 are phosphorylated downstream of TGF-β/BMP receptor signaling and translocate to the nucleus (Bourgeois et al. 2013; Lin et al. 2005; Osada et al. 2003; Pan et al. 2005; Raju et al. 2003). Once inside the nucleus, the C-terminus of MAN1 binds to Smads 2 and 3 and sequesters them at the nuclear envelope. This interaction is thought to compete for binding with transcription activator complexes, thereby regulating expression of gene downstream of TGF-β signaling.

In humans, several mutations have been described in MAN1 that lead to defects in the bone and skin (Hellemans et al. 2004). To our knowledge, no mutations have been described that result in skeletal or cardiac myopathy. However, several studies have been performed to uncover the role of MAN1 in model organisms. Global deletion of MAN1 using a genetrap approach led to embryonic lethality at E10.5 in mice. This was likely due to aberrant TGF-β signaling and vasculogenesis in the embryonic yolk sac (Ishimura et al. 2006; Cohen et al. 2007). Further analysis of the mutant hearts revealed pericardial edema in 31 of 32 embryos and defects in left-right axis formation of the heart (Ishimura et al. 2008). Further adding to a potential role of MAN1 in the heart, knockdown of MAN1 using a morpholino approach in *Xenopus* also led to cardiac defects (Reil and Dabauvalle 2013). Whether these findings were due to altered cardiomyocyte function or other confounding factors such as knocking down MAN1 in all tissues remain to be explored. Nevertheless, the expression of MAN1 during early stages of heart development makes studying MAN1 function in cardiomyocytes an interesting prospect.

## LEM domain-containing 2

LEM domain-containing 2 (Lem2) localizes to the INM and interacts with heterochromatin, A-type lamins, and BAF (Brachner et al. 2005; Cai et al. 2001, 2007; Liu et al. 2003). Lem2 is widely expressed in adult human and mouse tissues, with levels enriched in both the heart and skeletal muscle (Brachner et al. 2005; Chen et al. 2006) Similarly to MAN1, Lem2 contains an N-terminal LEM domain followed by a TM domain and a MSC domain at its C-terminus. Both LEM and MSC domains are reported to bind to heterochromatin in yeast (Barrales et al. 2016). These domains are highly conserved, suggesting they play an important role in Lem2 function. Lem2 interacts with the repressive histone modifications H3K9Me2 and H3K9Me3 in nematodes (Towbin et al. 2012), and in yeast, the loss of Lem2 led to the reduction of H3K9Me2, suggesting that it may play a role in establishing or maintaining this repressive mark (Barrales et al. 2016). As well as interacting with histone modifications, the LEM domain binds to the chromatin protein BAF, which itself directly binds to double-stranded DNA and directly to histones and influences histone modifications (Margalit et al. 2007; Montes de Oca et al. 2011, 2005, 2009; Tapia et al. 2015). Given the localization of Lem2 to the INM and proximity to heterochromatin as well as its interaction with A-type lamins, these properties make Lem2 a good candidate for regulating gene expression. In support of this, Lem2 has been shown to regulate ERK signaling in mouse myoblasts (Huber et al. 2009), is critical for nematode development (Barkan et al. 2012; Liu et al. 2003), and was found to be important for skeletal muscle regeneration after cardiotoxin injury (Tapia et al. 2015).

In humans, an autosomal recessive mutation in the LEM domain of Lem2 was recently identified that substitutes leucine 13 to arginine and leads to juvenile cataracts (Boone et al. 2016). Notably, some of these patients also died from sudden cardiac death; however, the cause of this was unclear and warrants further investigation.

In terms of mouse models to investigate Lem2 function, global loss of Lem2 resulted in embryonic lethality between E10.5 and E11.5. Although many normal hallmarks were observed in E10.5 knockout embryos, most of the tissues were substantially smaller in size. Furthermore, multiple MAP kinase pathways were upregulated, including ERK1/2, JNK, and p38 as well as AKT. As noted earlier, MAN1 KO mice display a similar timepoint of lethality at E10.5. However, the Lem2 KO embryos show a distinct phenotype from the MAN1 KO with no defects in vasculogenesis of the yolk sac, or changes in TGF-β signaling. Furthermore, Lem2 lacks the region used by MAN1 to interact with Smads (Caputo et al. 2006).

## Luma

Luma, alternatively known as transmembrane protein 43 (TMEM43), is associated with the LINC complex and was initially identified with a proteomics screen for INM proteins in neuroblastoma cells (Dreger et al. 2001). Luma is widely expressed in tissues including the heart (Bengtsson and Otto 2008; Dreger et al. 2001; Schirmer et al. 2003; Stroud et al. 2018) and is highly conserved throughout metazoans. It contains four TM domains and localizes to the INM, where it interacts with lamins A/C, B1, Emerin, and SUN2 (Bengtsson and Otto 2008; Liang et al. 2011).

Several mutations in Luma have been identified as the cause of arrhythmogenic cardiomyopathy (AC) (Christensen et al. 2011; Haywood et al. 2013; Hodgkinson et al. 2013; Honda et al. 2016; Merner et al. 2008; Milting et al. 2015). The best characterized is a missense, autosomal dominant mutation that changes serine 358 to leucine and was identified as the unequivocal cause of arrhythmogenic right ventricle cardiomyopathy type 5 (ARVC5) in humans (Christensen et al. 2011; Hodgkinson et al. 2013; Merner et al. 2008). The S358L mutation is in the third TM domain of Luma and was originally mapped in an extended eight-generation family in Newfoundland, Canada. The S358L mutation was subsequently identified in a German family, which shares a common haplotype with those from Newfoundland, suggesting that the mutation originated in Europe (Milting et al. 2015).

ARVC5 is a fully penetrant, lethal, sex-influenced disorder, in which men have a median lifespan of 41 compared to 83 in the control group and women have a median lifespan of 71. The disorder is fully penetrant at the age of 63 and 76 in men and women, respectively. The prominent features of ARVC5 are ventricular tachycardia, fibrofatty replacement of cardiomyocytes, premature ventricular contractions (PVCs), and left ventricular dilation resulting in heart failure and sudden cardiac death. The available therapies for ARVC5 patients are limited and include the use of implantable cardioverter defibrillator (ICD) devices. ICDs have shown to be more effective in ARVC5 patients particularly when used in men as a primary prophylaxis (before incidence of sustained ventricular tachycardia) versus secondary prophylaxis (after sustained ventricular tachycardia) (Hodgkinson et al. 2016).

Luma function was originally assessed in vitro using cardiomyocytes and non-cardiomyocytes (Franke et al. 2014; Jiang et al. 2017; Rajkumar et al. 2012; Siragam et al. 2014). However, there was limited information as to the role of Luma is cardiac development and function in vivo. Therefore, we took genetic approaches to uncover the role of Luma in cardiac function by generating several novel mouse models (Stroud et al. 2018). Using a series of cell-type specific antibodies and indicator mouse lines, Luma was expressed sporadically in cardiomyocytes, pericytes, and endothelial cells, whereas it was highly and uniformly expressed in fibroblasts and vascular smooth muscle cells (VSMCs). Furthermore, Luma localization was consistent between mouse and human myocardium as human myocardium from non-failing adults showed the same localization to the nuclear envelope. Importantly, the specificity of the Luma antibody was validated using Luma KO mouse tissue for both Western blots and immunofluorescence.

Because Luma was found in most cardiac cell types, a global knockout approach was taken to try and understand Luma’s role in heart function. Given the strength of data suggesting that Luma as the unequivocal cause of AC in humans, it was surprising that Luma KO mice appeared normal and had no abnormalities in cardiac function at baseline up to almost 2 years of age. Furthermore, no changes in the fetal gene programme or pro-fibrotic genes were observed, further supporting the notion that Luma KO hearts were normal. We hypothesized that other LINC complex proteins may compensate for loss of Luma, but the localization and levels of lamins A/C, B1, Emerin, Sun1, and Sun2 were unchanged (Stroud et al. 2018).

The LINC complex has been suggested to play a role in mediating response to hypertrophic stimuli (Cupesi et al. 2010; Stubenvoll et al. 2015); therefore, Luma KO mice were challenged with pressure overload using TAC. Similarly to their wildtype littermates, Luma KO mice displayed a normal hypertrophic response at 1–2 weeks, then progressively went into failure by 4 weeks post-TAC. Furthermore, the contractile function of Luma KO mice was unaffected in response to beta-adrenergic stimulation.

From these data, we concluded that ablation of Luma does not affect cardiac development or function, and that the S358L mutation that causes ARVC5 might be a gain of function mutation. To test this hypothesis, Luma S358L knock-in mice were generated using CRISPR/Cas9 technology to mimic the mutation described in humans with ARVC5. Mice heterozygous for the S358L mutation displayed no changes in cardiac function up to 1 year of age. Other mouse models of human autosomal dominant mutations exhibit a phenotype when both alleles are mutated in mice (Arimura et al. 2005; Mounkes et al. 2005). In contrast to other models, cardiac function in Luma S358L homozygous mice was normal and mutant Luma remained localized to the NE. A previous report suggested that Luma expression was downregulated in human myocardium from patients with the S358L mutation (Christensen et al. 2011). However, immunofluorescence and Western blotting revealed no changes between wildtype and both S358L heterozygous and homozygous mice.

An intriguing finding from this study was that both in human and mouse myocardium, Luma is predominantly expressed in fibroblasts and VSMCs. ARVC is normally considered a ‘disease of the desmosome’ (Zhang et al. 2015); therefore, it is unusual for a protein that is expressed at low levels in cardiomyocytes to result in ARVC. We therefore speculate that paracrine crosstalk between fibroblasts, VSMCs, and cardiomyocytes might be perturbed by the Luma mutation in humans. However, the pathophysiological mechanisms that occur as a result of the S358L mutation remain unclear. Because patients with the mutation develop ARVC5 later in life, we speculated that aging the S358L mice for longer might be necessary to elicit a phenotype. Alternatively, stressing the S358L knock-in mice using approaches to induce physiological or pathological hypertrophy may be required, as was shown for Luma’s binding partner, Emerin (Stubenvoll et al. 2015). Another possibility is that other yet-to-be-identified mutation(s) exist in modifier genes that are within the vicinity of the Luma gene and are therefore inherited in the same manner. Identification of these will require whole genome sequencing.

## Nuclear lamins

Nuclear lamins were first described in the 1970s and are type V intermediate filaments that readily self-associate to form parallel coil-coil homodimers (Gerace et al. 1978). The homodimers assemble higher-order filamentous structures to form the nuclear lamina that resides beneath the INM. The major components of the nuclear lamina in cardiomyocytes are A-type lamins A and C (encoded by the LMNA gene) and B-type lamins 1 and 2 (encoded by LMNB1 and LMNB2, respectively) (Fisher et al. 1986; Hoger et al. 1990; Lin and Worman 1995; McKeon et al. 1986). Lamins share a similar structure that is comprised of an alpha helical rod that is abutted to non-helical globular domains at the N- and C-termini (Stuurman et al. 1998).

The LMNA gene is alternatively spliced to generate lamins A and C, which are the major isoforms found in cardiomyocytes (Fisher et al. 1986; McKeon et al. 1986). Expression of A-type lamins is ubiquitous and developmentally regulated as they are only detected in differentiated cells (Broers et al. 1997; Rober et al. 1989; Solovei et al. 2013). Conversely, B-type lamins are constitutively expressed and are found in multiple tissues (Worman and Courvalin 2000). For a comprehensive review of nuclear lamin discovery and characterization, we refer readers elsewhere (Gerace and Huber 2012).

Nuclear lamins have been shown to play multiple roles in the cell, which are mediated via interactions at the nuclear periphery and nucleoplasm (Prokocimer et al. 2009). They are important for maintenance of nuclear structural integrity, chromatin organization, gene expression regulation, nuclear positioning, and cytoskeletal organization (Andres and Gonzalez 2009; Broers et al. 2004; Dechat et al. 2008; Folker et al. 2011; Lammerding et al. 2004) and interact with a plethora of different binding partners that enable direct or indirect interaction with chromatin through histones, lamin B receptor, heterochromatin protein 1, Emerin, and BAF (Burke and Stewart 2013; Mattout-Drubezki and Gruenbaum 2003; Wagner and Krohne 2007; Wilson and Foisner 2010).

As intermediate filament proteins, lamins provide mechanical stability to the nucleus (Lammerding et al. 2004). In support of this, nuclei from lamin A/C-null mouse embryonic fibroblasts have increased nuclear deformation, defective mechanotransduction, and impaired viability under mechanical strain (Lammerding et al. 2004). These data fit with in vivo data from lamin A/C-null mice in which cardiomyocyte nuclei are misshapen (Nikolova et al. 2004). Lamin A/C levels have been shown to scale with collagen 1 levels in tissues, whereas lamin B levels remain constant (Swift et al. 2013). These data suggest that lamin A/C may act as a ‘mechanostat’ and its levels are regulated with regard to tissue stiffness. It is tempting to speculate that a more fibrotic and stiffer myocardium may have elevated lamin A/C levels. However, the results from meta-analysis of existing proteomic datasets from cardiac tissue was inconclusive as to whether this is the case (Cho et al. 2017). Further work using defined animal models of cardiac fibrosis that can be tightly controlled and manipulated will be required to understand if there is a correlation between fibrosis and lamin A/C levels in the heart.

As well as providing mechanical stability, lamin A/C also plays a role in responding to mechanical force stimulation. For example, lamin A/C hemizygous mice have an attenuated response to TAC-induced pressure overload and impaired activation of the mechanosensitive gene EGR-1 (Lammerding et al. 2004). Further evidence comes from MEFs derived from lamin A/C-null or LMNA^N195K/N195K^ mice that showed impaired nuclear translocation of the mechanosensitive transcription factor MKL1 (Ho et al. 2013). Cardiac sections from both mouse lines had significantly reduced fractions of cardiomyocytes with nuclear MKL1. MKL1 is a co-activator of SRF, which is a master regulator of genes encoding cytoskeletal proteins.

These data suggest that lamin A/C not only plays a mechanical role but also may regulate gene expression. In support of this, hearts from the LMNA^H222P/H222P^ mouse model for EDMD and cardiomyopathy have upregulated MAPK signaling pathways, ERK 1/2, JNK, and p38-MAPK (Muchir et al. 2007b, 2012). Notably, these signaling pathways are activated prior to the onset of cardiomyopathy, suggesting that targeting these pathways with inhibitors may ameliorate cardiac dysfunction in the LMNA^H222P/H222P^ laminopathy model. Indeed, beneficial effects to the heart have been observed in mice when treated with various inhibitors of these pathways (Muchir et al. 2009; Wu et al. 2011, 2010).

Other potential roles of lamins include regulating muscle differentiation by differential binding of lamina-associated domain (LAD) regions of chromatin (Perovanovic et al. 2016). LADs are typically gene-poor regions that are tethered to the lamina in a constitutive or facultative manner (Pickersgill et al. 2006; van Steensel and Belmont 2017). Little is known about LADs in the heart, but work from Eric Schirmer’s lab suggests that tissue-specific nuclear envelope transmembrane proteins may play a role in striated muscle differentiation by regulating LADs at the NE (Robson et al. 2016). Whether LADs play a similar role in the heart remain to be elucidated.

Since the identification of mutations in lamin genes that cause cardiac and skeletal myopathies in the late 1990s and early 2000s, coined ‘laminopathies’, the nuclear lamin field has received considerable attention using cellular and mouse models to try and uncover the pathophysiological mechanisms (Bonne et al. 1999, 2000; Fatkin et al. 1999; Worman 2012; Worman et al. 2009). Laminopathies present as a myriad of different disorders including DCM (Fatkin et al. 1999), heart-hand syndrome (Renou et al. 2008), autosomal dominant (AD) EDMD (Bonne et al. 1999), limb-girdle muscular dystrophy (Muchir et al. 2000), lipodystrophies (Shackleton et al. 2000), and LMNA-related congenital muscular dystrophy (Quijano-Roy et al. 2008). For an extensive list of laminopathies we refer readers here (Captur et al. 2018). With regard to cardiomyopathy, mutations in the LMNA gene account for 5–8% of patients with genetic DCM (Hershberger et al. 2013; McNally and Mestroni 2017). In contrast, no mutations in B-type lamins have been identified that lead to cardiomyopathy. This may be because the severity of the phenotype is not tolerated in mammals. Indeed, loss of function models of both lamins B1 and B2 in mice results in embryonic lethality (Coffinier et al. 2010; Padiath et al. 2006; Vergnes et al. 2004).

As mentioned above, many mouse models have been generated to understand the function of lamin A/C in the heart (Arimura et al. 2005; Cattin et al. 2013; Kubben et al. 2011; Lu et al. 2010; Mewborn et al. 2010; Mounkes et al. 2005; Nikolova et al. 2004; Sullivan et al. 1999; Wang et al. 2006). Interestingly, some of these mouse models (e.g. H222P, N195K) need to be homozygous for the LMNA mutation to elicit a phenotype, whereas in humans, the disease is normally dominant. This could be due to unidentified mutations in modifier genes or greater dosage sensitivity in humans. Thus far, no approaches have been taken to ablate lamin A/C, B1, or B2 expression in a cardiomyocyte-specific manner. This is somewhat surprising given the robust phenotypes observed with the global knockout mice. Combined with the data from the global knockout mice, these data would lend new insights on which cell-type expression of lamins A/C, B1, and B2 is critical for normal heart function. Furthermore, the original lamin A/C knockout mice produce a 54 kDa truncated version of lamin A/C that arises as a result of an unforeseen splicing event (Jahn et al. 2012; Nikolova et al. 2004). The truncated version contains the N-terminal globular domain and rod domains, whereas a large proportion of the C-terminal globular domain is missing. Therefore, generation of LMNA floxed mice would not only enable the original model to be validated as a loss of function model but also allow tissue-specific ablation.

## Future directions

The importance of the LINC complex in fundamental cellular processes has become apparent since the discovery of mutations in LINC complex-encoding genes that cause cardiomyopathy (McNally and Mestroni 2017; Meinke and Schirmer 2016; Mejat and Misteli 2010; Worman et al. 2010). The LINC complex structurally supports the nucleus and physically couples the nucleoskeleton to the cytoskeleton (Crisp et al. 2006; Sosa et al. 2013). Furthermore, it is hypothesized to act as a mechanosensor that translates mechanical stimuli into biochemical cues that mediate gene expression changes and affect chromatin organization (Jaalouk and Lammerding 2009; Lombardi and Lammerding 2011). It is clear that the structural and gene expression roles are not mutually exclusive and that modulating one aspect of LINC complex function will likely influence the other. As a consequence, disruption of the LINC complex and its associated proteins affects cellular function and contributes to cardiomyopathy as described in this review.

Integrated approaches that combine mouse models, cell-based assays, and biophysical analyses have contributed to our progress in understanding LINC complex function. Armed with the knowledge of the mutations in the LINC complex that cause cardiomyopathy, the next steps will be to uncover the pathophysiological molecular mechanism underlying disease.

As described in this review, many of the LINC complex and its associated proteins have been studied using global knockout mouse approaches, including Nesprins, SUN proteins, Emerin, LAP1, LAP2, MAN1, LEM2, Luma, and nuclear lamins. Therefore, a lot remains to be uncovered in terms of the tissue-specific roles in cardiomyocytes as well as the function of the different isoforms. Another challenge will be to identify the interaction networks of the LINC complex proteins. Until recently, this remained elusive, partly due to the insolubility of the nuclear lamina and chromatin (Roux et al. 2012). However, with the advent of proximity proteomic approaches, such as Bio-ID and APEX, the LINC complex interactome can now be explored (Kim et al. 2016; Kim and Roux 2016; Rhee et al. 2013; Roux et al. 2012). This approach has been mostly applied in cell culture, but mouse lines are currently being engineered to identify binding partners in an in vivo setting.

As well as taking these approaches, complementary assessment of LINC complex function in human cardiomyocytes generated using induced pluripotent stem cell (iPSC) technology will be essential. Indeed, next steps have been taken using this protocol from laminopathy patients (Shimojima et al. 2017; Siu et al. 2012). Despite the question of cardiomyocyte maturity in iPSC-derived cardiomyocytes, the field is rapidly progressing thanks to the technology to generate engineered heart tissue (Ronaldson-Bouchard et al. 2018; Zimmermann et al. 2006). Furthermore, the use of CRISPR/Cas9 machinery to correct patient mutations and generate isogenic controls will further strengthen this technology. Not only will this approach enhance our understanding of the fundamental biology of the LINC complex, it will enable the development of novel therapeutics to target the devastating effects of LINC complex-associated cardiomyopathies.

## Abbreviations

AC: Arrhythmogenic cardiomyopathy
CH: Calponin homology
DCM: Dilated cardiomyopathy
EDMD: Emery-Dreifuss muscular dystrophy
INM: Inner nuclear membrane
KASH: Klarsicht, ANC-1, and Syne homology
KO: Knockout
LAD: Lamina-associated domain
LAP: Lamina-associated peptide
LINC: Linker of nucleoskeleton and cytoskeleton
MEF: Mouse embryonic fibroblast
NE: Nuclear envelope
NPC: Nuclear pore complex
ONM: Outer nuclear membrane
PNS: Perinuclear space
SUN: Sad1/UNC-84
TAC: Transverse aortic constriction
TM: Transmembrane
VSMC: Vascular smooth muscle cell
